# Evolution of Nutritional Habits Behaviour of Spanish Population Confined Through Social Media

**DOI:** 10.3389/fnut.2021.794592

**Published:** 2021-12-16

**Authors:** Miguel Mariscal-Arcas, Sonia Delgado-Mingorance, Borja Saenz de Buruaga, Alba Blas-Diaz, Jose Antonio Latorre, Manuel Martinez-Bebia, Nuria Gimenez-Blasi, Javier Conde-Pipo, Leticia Cantero, Alejandro Lopez-Moro, Maria Jose Jimenez-Casquet

**Affiliations:** ^1^Department of Nutrition and Food Science, University of Granada, Granada, Spain; ^2^Master of Food and Fit, University of Granada, Granada, Spain; ^3^Department of Food Technology, Nutrition and Food Science, University of Murcia, Murcia, Spain; ^4^Department of Nutrition and Dietetics, Faculty of Health Sciences, Catholic University of Avila, Avila, Spain

**Keywords:** social media, database, nutrition methodology, population confined, diet

## Abstract

**Introduction:** In Spain, on 14 March 2020, a state of alarm is declared to face the health emergency situation caused by the COVID-19 coronavirus, limiting the freedom of movement of people. The Spanish population is confined.

**Objective:** With this situation, “NUTRITIONAL HEALTH IS NOT CONFINED” arises a research project that seeks to promote nutritional education based on the pattern of the Mediterranean diet (MD) using new computer technologies. It is about providing the population with the information of general interest about the promotion of a healthy diet through social networks and analysing the impact of its dissemination, in the form of a longitudinal intervention study of the Spanish nutritional evolution during confinement, with a daily survey format, and it is intended to assess food consumption during the period of confinement. Materials and methods: In total, 936 participants were asked every day. Short publications were published every day based on the scientific evidence (FAO, WHO, AECOSAN) through social media such as Instagram, accompanied by a questionnaire of 11 questions (yes/no) where it was intended to assess the evolution of daily consumption.

**Results and Discussion:** The diffusion through social media has allowed to have a greater reach of the population. We observed that mood throughout confinement generally improves. There are certain eating habits from the MD that are well established in the daily diet of our population, such as the consumption of fruits, vegetables, legumes, dairy products, and eggs. It seems that enjoying good health is a growing concern in pandemic situations, which is why inappropriate behaviours such as “snacking” between meals or the consumption of processed foods such as snacks, industrial pastries, soft drinks, and sweets are avoided, increasing the amount of healthy food such as meat and fish. This study opens up future avenues of research promoting MD and implements new cohort nutritional databases, especially about young adult people, who are adept at navigating digital spaces and therefore using social media.

## Introduction

Throughout history, there have been various catastrophes such as wars, terrorist attacks, floods, earthquakes, and pandemics, which highlight the vulnerability of the population and require the urgent mobilisation of interventions (1). In Spain, on 14 March 2020, a state of alarm is declared to face the health emergency situation caused by the COVID-19 coronavirus, limiting the freedom of movement of people. The Spanish population is confined. Faced with this situation and with a project already started on how social networks influence the dissemination of healthy aspects based on the Mediterranean diet (MD), “NUTRITIONAL HEALTH IS NOT CONFINED” (#nutritionalhealthisnotconfined) arises. A scientific initiative for the transfer of research aims to make confinement more bearable by promoting nutritional education through the daily publication in a digital blog, of posts based on scientific evidence, of different aspects related to a healthy diet. The dissemination of content on social media presents a series of advantages with respect to the paper format, such as immediate communication and instantaneous assessment of the reactions of readers, and also sharing knowledge of any field reaching more niches of population (2). That is why this format is chosen with the aim of reaching as many people as possible without biases of age, profession, interest in a particular healthy life, or level of training or special knowledge, only the employment of the social network chosen for the dissemination of #nutritionalhealthisnotconfined (3).

This blog would be based on the Mediterranean pattern for its great association with the idea of health and quality of life, and also for its proven beneficial effects on cardiovascular diseases and even prevention of some types of cancer. One of the components that characterise this eating pattern is olive oil, whose main fatty acid is oleic acid. Because it is also the main source of lipids, the MD has a high monounsaturated or saturated fatty acid (MUFA/SFA) ratio compared to other countries in the world that use animal-type fats, with the corresponding advantages at the cardiovascular level (4). In addition, the high consumption of foods of plant origin such as legumes, fruits (as a usual dessert), vegetables, mushrooms, and nuts produces a great contribution of fibre, vitamins, and minerals at the same time that they provide a large amount of water, drink par excellence in the Mediterranean and fundamental in our diet. In addition, it establishes a daily consumption of bread and cereals such as pasta and rice, preferably whole grains, and also dairy products (mainly yoghourt and cheeses) since they are a source of protein of high biological value, of vitamins and minerals (calcium, phosphorus), and of the well-known probiotics capable of improving the balance of the intestinal microbiota (5). As a source of protein, the Mediterranean pattern defends animal protein, but prioritising fish and eggs over meat. The MD not only focuses on food, but also defends a healthy lifestyle, which is why it emphasises the need to perform physical activity every day adapted to our abilities to maintain good health (6, 7).

As usually happens with any natural catastrophe, both physical and psychological consequences remain in the population that must be analysed and treated (1). When disaster strikes, everyday life patterns and normal behaviour are disrupted. In the case of confinement, one can speak of loneliness and social isolation, which in recent years the World Health Organisation (WHO) had classified as a challenge for global public health. Increased stress and anxiety due to the obligation to be locked up, due to the loss of routine activities, due to the lack of information about the situation experienced or the uncertainty of what will happen in the near future, attached at fear of possible contagion, and also the employment and economic situation of each person, can be some of the most common manifestations (8). In addition, they can affect not only on a psychological level, but also on a physical level, compromising our immune system due to a chronic inflammatory reaction (9). Maintaining the mental health of citizens has been a challenge these days, which is why telephone medical consultations and online psychological counselling services have been enabled. In this case, communication and social media have done a very positive job: being able to communicate and even see loved ones and friends who are far away or even read news about how to cope with confinement (10).

For all this, the ultimate goal of “NUTRITIONAL HEALTH IS NOT CONFINED” (#nutritionalhealthisnotconfined) is proposed as a measure to help the population during confinement, where to explain the properties of the foods that make up the MD, some original recipes how to consume them, their health benefits, and even how they can affect our declared mood. Social media and new technologies can be the good tools (3). Therefore, once the scientific literature has been reviewed, and the insufficiency of studies and the inconsistency of results have been proven, the main objective of this applied research work is to provide the population with information of general interest on the promotion of a healthy diet through social media and analyse the impact of its dissemination, in the form of a longitudinal intervention study of the Spanish nutritional evolution during confinement. In a daily survey format, designed and validated by authors (3, 6, 7, 11–14), it is intended to assess food consumption during the quarantine period. Databases and information achieved through this new format of study could be useful to inform the population through social media about forms of healthy eating based on the MD, publicise not so widespread foods or different ways of consuming them and also their benefits in the body, describe possible mood changes during confinement and how to cope with them through diet, explain different aspects related to a healthy life style and physical activity practise, carry out daily surveys to assess the way the population feeds during quarantine and also making an assessment of the declared daily mood and body perception of the participants, and analysing how all of the above is disseminated through something newer than paper format (magazines, newspapers, and television) such as social media and how it this new format impacts the population.

## Materials and Methods

The format of “NUTRITIONAL HEALTH IS NOT CONFINED” (#nutritionalhealthisnotconfined) was used through publications of short duration and free dissemination based on the transfer knowledge of the authors using international recommendations FAO/WHO (15–18) and scientific evidence, disseminating this information and asking everyday through Instagram social media. Daily questionnaire included written text and publications in video format. Each daily questionnaire was accompanied by 11 questions with dichotomous answer options (yes/no) where it was intended to describe and assess the evolution of daily consumption of water, alcohol, vegetable products, animal products, processed and ultraprocessed, and those related to nutritional habits as snacking between meals or other important as changes in daily body perception, changes in mood perception and the influence that each daily questionnaire may had had on the subject.

### Study Population

In total, 936 subjects were freely recruited and surveyed each day during the quarantine state occurred in Spain from March to July 2020. We used the official social media (Instagram) of a Spanish winter sports federation (@fadiandalucia) as representative sample of the physically active Spanish population who have more than 1,000 people federated in Spain and more than 2,200 followers. We also used the official social media (Instagram) of scientific knowledge transfer academic platform designed by our nutritional research group in the Department of Nutrition and Food Science of the University of Granada, Spain call MM Health Science (@mmhealthscience) [section: “#lasaludnutricionalnoseconfina” (#nutritionalhealthisnotconfined)] (3) with more than 820 followers. The validity and reliability of this tool used in this study were obtained through the research work published by Mariscal-Arcas (3, 6, 7, 11–14). The age of the subjects who voluntarily participated in the study was between 17 and 65 years, excluding any subject under 17 years of age from the study. Only followers of one of these two official social networks were allowed to participate to control the population. The study was approved by the Research Ethics Committee of the Andalusian Public Health Service, Spain (reference number. 0756-N-20). The research protocol was carried out in accordance with the Declaration of Helsinki for Human Studies of the World Medical Association, with strict respect for the confidentiality of the information in accordance with Organic Law 15/1999, of 13 December, on the protection of personal data in all the processes of collection and treatment of the information obtained and Organic Law 3/2018, of 5 December, on the protection of personal data and guarantee of digital rights. The population under study is all followers of mentioned accounts, who participated voluntarily by consenting through a private request on their profiles, as represented in Table 1. To analyse dietary changes during confinement, previous consumption data for each food group according to the SENC Healthy Eating Guidelines have been used (19).

**Table 1:** Population that constitutes the study by age groups and origin as a percentage (*n* = 936).

			**@fadiandalucia**	**@mmhealthscience**		
			**% (*N*)**	**% (*N*)**		
Sex	Men		62 (427.80)	37 (91.02)		
	Women		38 (262.20)	63 (154.98)		
Age range (yrs)	17–24		12 (76.90)	20 (47.74)		
	25–34		28 (195.20)	46 (115.16)		
	35–44		29 (200.10)	21 (52.66)		
	45–54		20 (139.00)	8 (20.68)		
	55–64		7 (49.30)	8 (20.68)		
	≥65		2 (14.80)	1 (3.46)		
Origin	Granada		40 (376.00)	20 (186.10)		
	Madrid		18 (169.00)	1.8 (17.22)		
	Malaga		2.2 (20.70)	0 (0.00)		
	Barcelona		1.4 (13.80)	0 (000)		
	Seville		1.4 (13.80)	0 (0.00)		
	Murcia		0 (0.00)	13.3 (124.60)		
	Cangas		0 (0.00)	0.7 (7.38)		
	Lorca		0 (0.00)	0.7 (7.38)		
Declared co-morbidities	Respiratory diseases		4.2 (39.31)	4 (37.44)		
	Cardiovascular diseases		0 (0.00)	1.6 (14.97)		
	Diabetes		1.9 (17.78)	1 (9.36)		
	Hypercholesterolemia		2.3 (21.52)	3 (28.08)		
	Hypertension		2 (18.72)	2.1 (18.72)		
	Allergies/intolerances		1.4 (13.10)	2.7 (25.27)		
		**Mean (SD)**	**Min**	**Max**	***P*** ^*^	***P*** ^**^
Age (yrs)	Men	34.78 (10.92)	19.00	65.00	0.706	
	Women	35.40 (12.48)	17.00	72.00		
	Total	35.21 (12.00)	17.00	72.00		
Weight before confinement (Kg)	Men	80.43 (11.82)	53.00	114.30	0.001	
	Women	63.64 (11.10)	41.00	98.90		
	Total	68.85 (13.72)	41.00	114.30		*R* = 0.995 *P* = 0.001
Weight at the end of confinement (Kg)	Men	80.43 (11.82)	53.00	114.30	0.001	
	Women	63.64 (11.10)	41.00	98.90		
	Total	68.85 (13.72)	41.00	114.30		
Height (cm)	Men	178.16 (6.94)	168.00	196.00	0.001	
	Women	164.88 (6.13)	148.00	181.00		
	Total	169.00 (8.87)	148.00	196.00		
BMI before confinement (Kg/m^2^)	Men	25.29 (3.15)	18.56	36.57	0.001	
	Women	23.43 (4.10)	15.70	38.67		
	Total	24.01 (3.92)	15.70	38.67		*R* = 0.993 *P* = 0.001
BMI at the end of confinement (Kg/m^2^)	Men	25.43 (0.37)	18.56	36.57	0.001	
	Women	23.39 (0.32)	16.04	38.67		
	Total	24.02 (0.25)	16.04	38.67		

* *Student's t-test by gender*.

** *Pearson's correlations with the Total variable*.

The material used for the development of the publications went from official database sources of information in the field of nutrition (FAO, WHO) and from scientific sources based on evidence (PubMed, Scopus, and Google Scholar). To evaluate the results, the percentage of affirmative responses in the survey is analysed, which associates it with the days on which the study is carried out. As a strength, this nutritional study through social media allows to have a wider scope of population including different points of Spain distant from each other and mostly young adult population, however, part of the limitations of the study lies in the insufficient scientific work that currently exists. Another limitation in the use of surveys on the Instagram platform is the impossibility of adding many extended questions.

### Statistical Analysis

SPSS version 22.0 (IBM. Chicago. IL) was used for the statistical analysis. A descriptive analysis was conducted to calculate means, standard deviations (SD), medians, maximum and minimum values, histograms, and normality tests, followed by the application of the Student's *t*-test, analysis of variance (ANOVA), Pearson's correlations. *p*-Value < 0.05 was considered statistically significant.

## Results

### Usual Eating Habits

Before the state of alarm and confinement, the Spanish population, according to the SENC, has the following eating habits: 97% of people regularly consume meat, at levels above the recommendations for red and processed meats in 57% of cases, especially in those under the age of 35. In total, 95.5% of people usually consume fish, 98.8% eggs, and 88.6% dairy products. Only 30% meet the recommendations for fruit consumption and 21.3% for vegetables, although 55% consume at least one daily serving of whole fruit and 53.7% at least one serving of vegetables. A significantly higher proportion of women have adequate vegetable intakes. In total, 54% of people report usual intakes of pulses in line with recommendations, although 51% of those over 55 years of age report inadequate intakes for this food group, as for cereals and potatoes. A total of 89.6% of people report using virgin olive oil is an added fat in culinary preparations, whereas 20.4% of people also use olive oil and 16.8% sunflower oil. As regards the consumption of sweet and bakery products, 84% of respondents reported regular consumption, although 75% of men and 65% of women consumed them occasionally. Those under the age of 35 years have a higher proportion of inadequate consumption for this food group (40%), for savoury snacks (39%), and sugary drinks (10%) (19).

**Figure 1:**
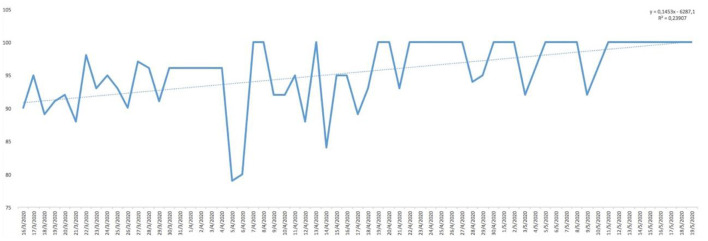
YES percentage response analysis of did you drink water yesterday? Depending on the days of study.

**Figure 2:**
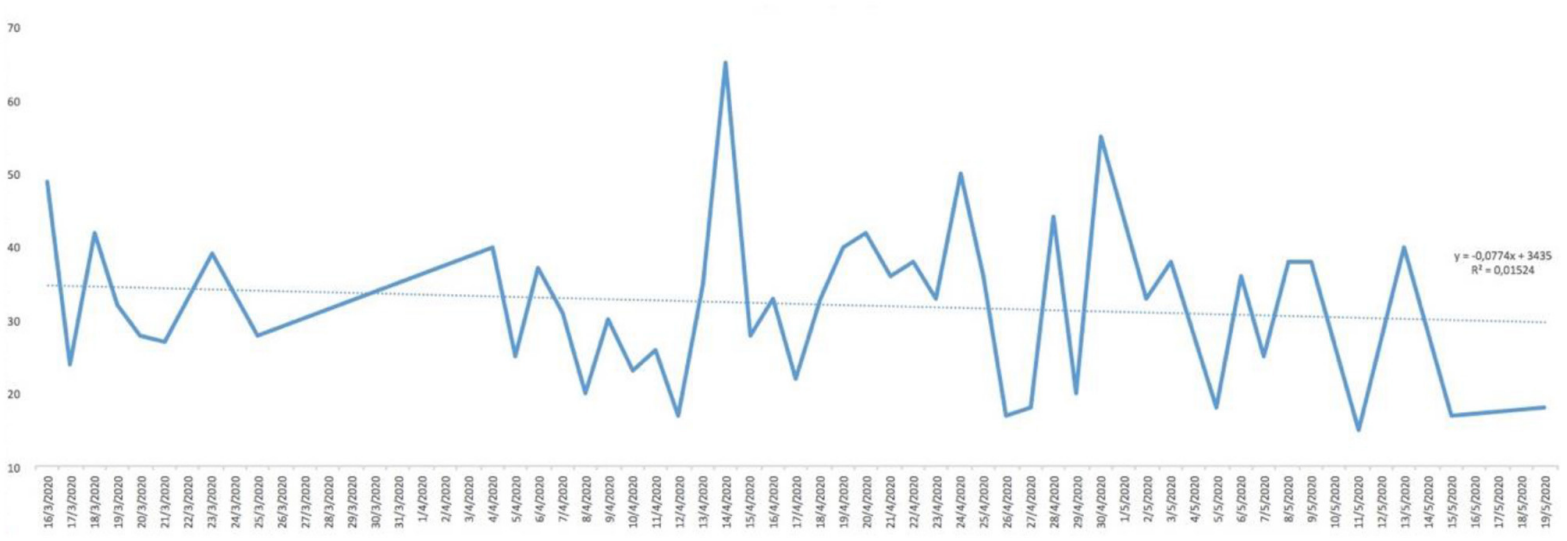
YES percentage response analysis of did you drink alcohol? Depending on the days of study.

**Figure 3:**
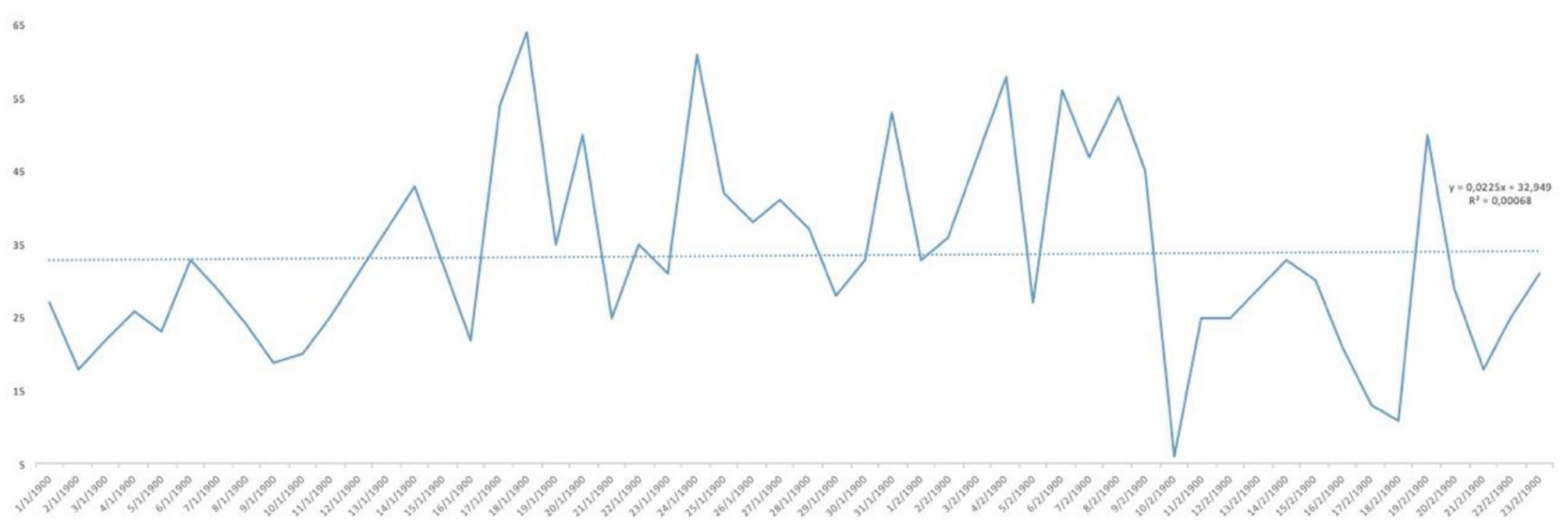
YES percentage response analysis of Do you feel heavier than yesterday? Depending on the days of study.

**Figure 4:**
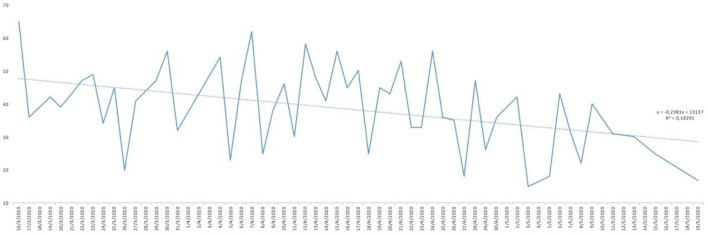
YES percentage response analysis of did you sting between hours? Depending on the days of study.

**Figure 5:**
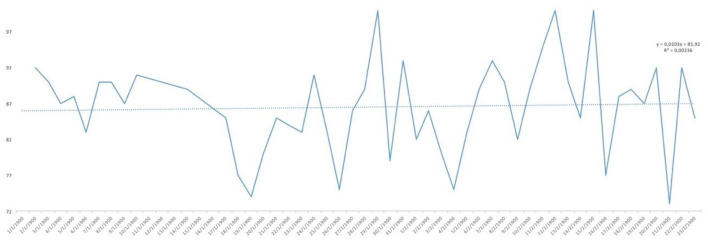
YES percentage response analysis of did you consume any dairy or milk yesterday? Depending on the days of study.

**Figure 6:**
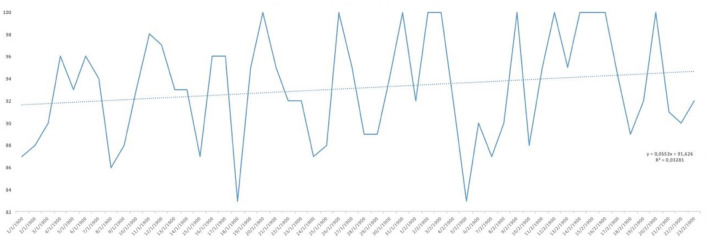
YES percentage response analysis of were there eggs in any of your meals? Depending on the days of study.

**Figure 7:**
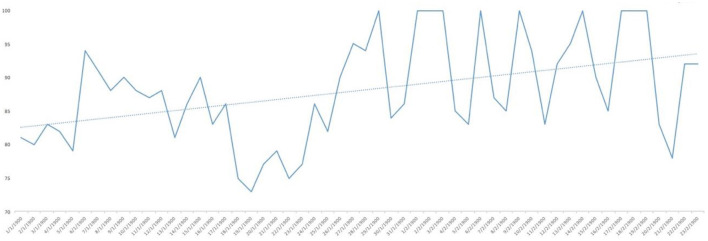
YES percentage response analysis of did you eat fruit, vegetables or legumes yesterday? Depending on the days of study.

**Figure 8:**
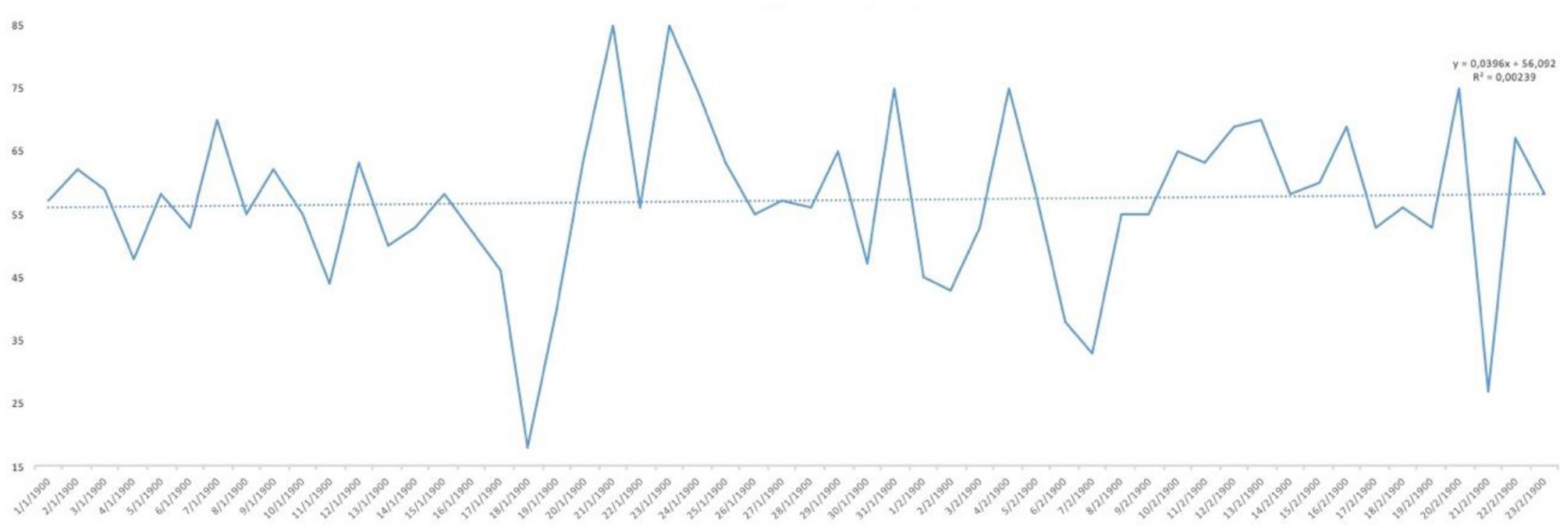
YES percentage response analysis of did you consume any snack, sauces, sweets, industrial pastries or soft drinks? Depending on the days of study.

Analysing the result of the percentage of ‘YES' responses for each of the responses in relation to the days of the study, trend lines were generated for each of the items asked. As can be seen in Figure 1, the trend in water consumption, which turned out to be quite high, is not falling below 90% except for specific days that are also concentrated in the first half of the study and then remaining around 100% quite consistently. However, analysing the trend line of alcohol consumption, as can be seen in Figure 2, it remained constant throughout the entire confinement, with a mean of “YES” responses not more than 35%. On the other hand, in the case of the answers to the question “Do you feel heavier than yesterday?”, the trend line of the graph was slightly downwards from 30 to 40% (Figure 3). Regarding the “snacking” between hours, the situation was totally different, since there was a clear decrease as the days passed, decreasing from approximately 50 to 30% (Figure 4). The consumption of foods of animal origin showed very different results from each other. In the case of dairy products (Figure 5), the percentage of YES responses to the question Did you consume any dairy or milk? remained around 85% throughout the confinement time with a slight upward trend. However, the consumption of animal protein sources such as meat and fish rose to 90–95% toward the second half of the study with several days of 100% affirmative responses. Regarding daily consumption of egg, the trend line remained practically straight around 50–55% of positive responses as shown in Figure 6. Foods such as fruits, vegetables, and legumes (Figure 7) were consumed in a percentage above 90% throughout the entire quarantine, with a slight upward trend. However, the consumption of snacks, sauces, sweets, industrial pastries, or soft drinks fell from 55% to 35% as the progress of the study days (Figure 8).

**Figure 9:**
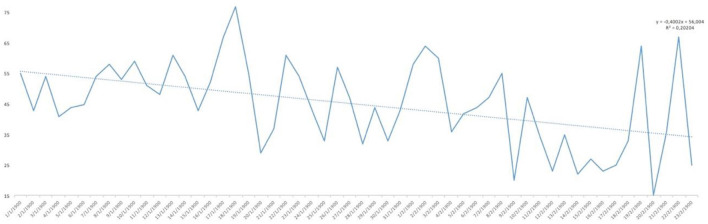
YES percentage response analysis of does your mood improve? Depending on the days of study.

**Figure 10:**
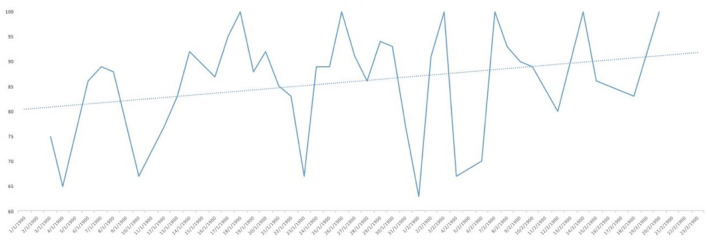
YES percentage response analysis of has this post been useful for your health today? Depending on the days of study.

In Figure 9, the affirmative responses regarding mood are reflected and, although we see large peaks up and down, the trend line was slightly upwards, reaching 55% positive responses. Finally, as to whether the posts published are useful for the current health of the population, as can be seen in Figure 10, the analysis of positive responses began to be high (80%), increasing as the days of confinement passed in general, although with great diversity in the responses as shown by the peaks in the graph.

**Figure 11:**
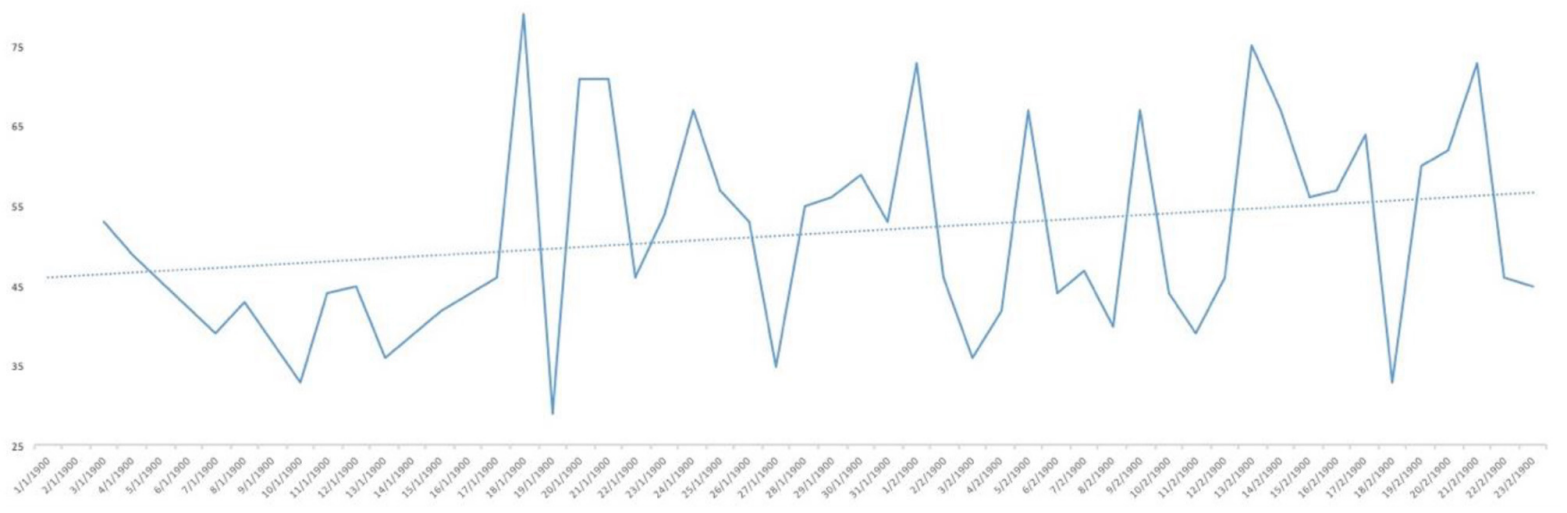
YES percentage response analysis of did you eat any meat or fish? Depending on the days of study.

In Table 2, descriptive statistical parameters are analysed, such as the minimum and maximum values of positive and negative responses in percentage, the mean and median of these responses, and the standard deviation (SD). The affirmative answer to the question “Do you feel heavier than yesterday?”(Figure 11) is, at some point, presented a maximum of 65%, while in the case of the negative answer, it was 85%. However, the mean of the positive answer corresponds only to 32.15% and 67.84% for the negative answer. The consumption of water showed results of more positive than negative responses. At some point, 100% affirmative responses were declared with a minimum of 79%. In this case, the mean and median correspond to 95% [mean = 95.05% (SD: 5.29) and median = 95%]. The “pecking” between hours declared more disparate responses with a maximum of 65% affirmative responses and a minimum of 15%. The mean and median are not coincident [mean 38.92% (SD: 12.21) and median 41%]. The negative response for alcohol consumption presented higher maximum (94%) and minimum (36%) than for the positive response. However, there were not very uniform results with the mean of 66.42% (SD: 13.65); it should be noted that the median does not have similar value as the mean. In the case of the consumption of fruit, vegetables, and legumes, the percentage of YES remained high throughout the study, since the minimum of positive responses was 83%, which means that only almost 20% in at some point declared not to consume fruit, vegetables, and legumes. The results regarding dairy consumption presented a maximum of 100% affirmative responses and a minimum of 73%. These responses were more uniform among the study participants showing a mean of 86.52% (SD: 6.46) and a median of 87% practically equal to the value of the mean. At some point, a maximum of 85% of the participants declared consuming eggs in their meals, but on the other hand, there was also a maximum of 82% negative responses regarding the consumption of eggs and although mean and medians coincide, presenting a SD > 12 for both cases. The answer to the question Did you eat meat or fish yesterday? (Figure 11) is that the total of the responses leaned toward the YES, reaching 100% [mean = 88.03% (SD: 7.77)] in some cases compared to 25% in the case of negative responses [mean = 11.74% (SD: 7.56)]. However, for the consumption of snacks, sweets, industrial pastries, and soft drinks, the results were more controversial. Regarding maximum and minimum, both the affirmative and negative responses reached high values throughout the confinement time (positive: 77% and negative: 85%). For their part, the mean and median remain at almost identical values (45 and 44.5%, respectively, for YES and 55 and 55.5%, respectively, for NO) but the SD with respect to the mean is higher than in other answers (SD: 14.01). The state of mind of the study participants throughout the days, analysing Table 2, shows a maximum of 79% affirmative responses compared to a maximum negative 71%, so there was disparity in the results as evidenced by the SD: 12.79 from the mean. Concluding the analysis of Table 2, regarding the question Has today's post served you?, the affirmative responses throughout the days were more common (on 1 day of the study duration a 100% YES was reached) with a mean that is 85.65% (SD: 10.56) and median of 88%.

**Table 2:** Percentages of responses for each of the study questions.

	**Minimum**	**Maximum**	**Mean**	**Median**	
	**%**	**%**	**%**	**SD**	**%**
Yes I feel heavier than yesterday	15..00	65.00	32.15	10.89	33.00
I don't feel heavier than yesterday	35.00	85.00	67.84	10.89	67.00
Yes I drank water all day yesterday	79.00	100.00	95.05	5.29	95.00
I didn't drink water all day yesterday	0.00	21.00	4.94	5.29	5.00
Yes, I ate between hours yesterday	15.00	65.00	38.92	12.21	41.00
I did not eat between hours yesterday	35.00	85.00	61.07	12.21	59.00
Yes, I drank alcohol yesterday	6.00	64.00	33.58	13.65	31.00
I didn't drink alcohol yesterday	36.00	94.00	66.42	13.65	69.00
Yes I had fruit, vegetables, vegetables or legumes yesterday	83.00	100.00	93.14	4.80	93.00
I did not have fruit, vegetables, vegetables or legumes yesterday	0.00	17.00	6.77	4.83	7.00
Yes I had milk or some dairy yesterday	73.00	100.00	86.52	6.46	87.00
I did not drink milk or any dairy yesterday	0.00	27.00	13.47	6.46	13.00
Yes there were eggs at any meal yesterday	18.00	85.00	57.18	12.79	57.00
There were no eggs at any meal yesterday	15.00	82.00	42.62	12.94	43.00
Yes I had meat or fish yesterday	73.00	100.00	88.03	7.77	87.00
I did not have meat or fish yesterday	0.00	25.00	11.74	7.56	12.50
Yes I had snacks, sauces, sweets, pastries or soft drinks yesterday	15.00	77.00	45.00	14.01	44.50
I did not have snacks, sauces, sweets, pastries or soft drinks yesterday	23.00	85.00	55.00	14.01	55.50
Yes it improves my mood	29.00	79.00	52.00	12.79	49.00
Doesn't improve my mood	21.00	71.00	48.00	12.79	51.00
Yes, today's post has helped me	63.00	100.00	85.65	10.56	88.00
Today's post has not served me	0.00	37.00	14.35	10.56	12.00

**Figure 12:**
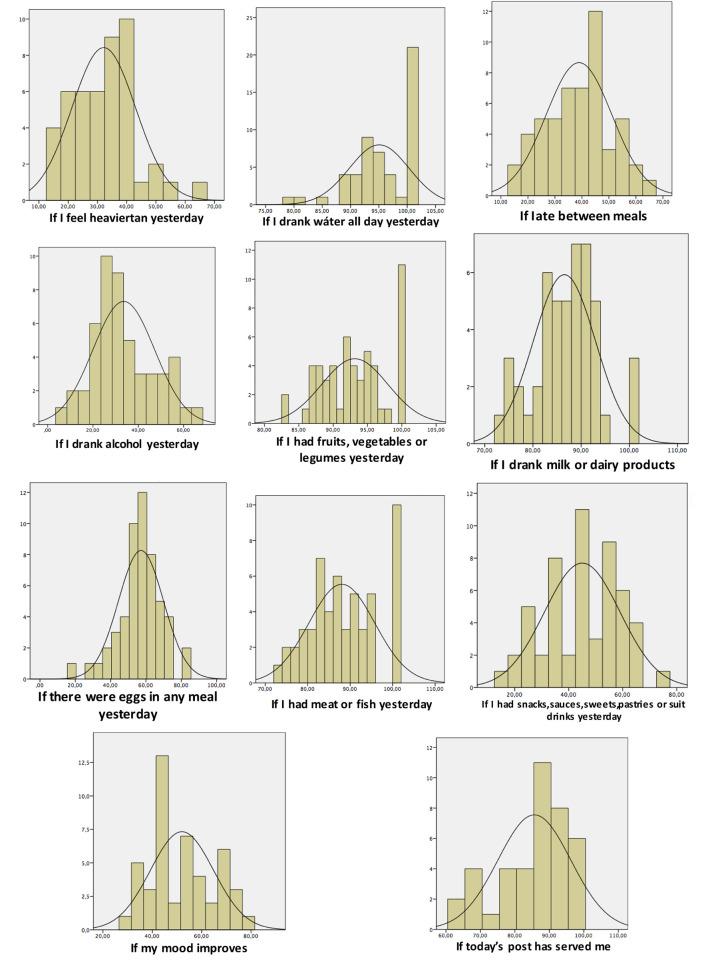
Normality curves for each of the questions throughout the study period.

Normality tests: The analysis in the form of histograms for each of the positive responses of the survey establishes symmetric normality curves of normal distribution in all of them (Figure 12). Some of them are flatter, as in the case of the consumption of water or fruit, vegetables, and legumes whereas others such as the consumption of dairy or eggs appear more elongated.

*Pearson's correlation analysis between variables:* *Table 3* *refers to the correlation between the two variables analysed for each question: percentage of affirmative responses and the days of confinement. That is, it confirms whether the results presented in the previous graphs are significant or not, whether there is a correlation between them or not. Pearson's correlation coefficient was used with a statistical significance level of p* ≤ *0.05*. Only some type of correlation was observed for the responses on water consumption, snacking between meals, consumption of meat and fish, and consumption of snacks, sauces, sweet pastries, or soft drinks. The existing correlation for water consumption with respect to the days on which the study was *R* = 0.489 in the coefficient with a significance level of 97% (*p* = 0.001), for which a positive correlation is declared. As the days passed, the consumption of water increased. Regarding the “pecking” between hours, the opposite case occurs: a negative correlation is observed; As the days passed, their consumption decreased as indicated by a negative Pearson's coefficient (*R* = −0.428, *p* = 0.001). The same case is the consumption of snacks, sauces, sweets, industrial pastries, and soft drinks, obtaining a correlation of *R* = −0.444, *p* = 0.001. Of all the data analysed, the last one that presented correlation was the question related to the consumption of meat or fish, obtaining in this case a positive correlation (*R* = 0.397, *p* = 0.003), for which an increase in this type of food as the days of confinement progress.

**Table 3:** Correlations in the evolution of the percentages of affirmative responses during the study period.

	**Evolution of days**	
Yes I feel heavier than yesterday	Pearson's correlation	−0,123
	*P*	0.414
Yes I drank water all day yesterday	Pearson's correlation	0.489**
	*P*	0.001
Yes I ate between hours yesterday	Pearson's correlation	−0.428**
	*P*	0.001
Yes I drank alcohol yesterday	Pearson's correlation	0.031
	*P*	0.832
Yes I had fruit, vegetables, vegetables or legumes yesterday	Pearson's correlation	0.174
	*P*	0.209
Yes I had milk or some dairy yesterday	Pearson's correlation	0.030
	*P*	0.839
Yes there were eggs at any meal yesterday	Pearson's correlation	0.047
	*P*	0.738
Yes I had meat or fish yesterday	Pearson's correlation	0.397**
	*P*	0.003
Yes I had snacks, sauces, sweets, pastries or soft drinks yesterday	Pearson's correlation	−0.444**
	*P*	0.001
Yes it improves my mood	Pearson's correlation	0.235
	*P*	0.112
Yes, today's post has helped me	Pearson's correlation	0.284
	*P*	0.076

## Discussion

Digital nutrition promotion interventions provide an opportunity to address the public health issue of improving people's nutrition (3, 20). There are inherent elements of subjectivity in the interpretation of this case study (3, 21); however, it presents insight into how our subjects (audience) engaged with one science communication endeavour using digital platforms as social media. Zarnowiecki found a positive effect of the digital intervention on child nutrition across a range of dietary outcomes (20). Additionally, having reviewed and analysed the data obtained in Instagram, we consider these data to be of great use for future research related to the implementation of MD, and also to promote nutritional health through social media, applications, blogs, etc. (3, 22–24, 24). The main objective of this study was to provide the population with the information of general interest on the promotion of healthy eating through social media and analyse the impact of its dissemination, in addition to assessing the consumption of food during the period of confinement. The results suggest that a high percentage of the population considered the action positive as indicated by Mariscal-Arcas (3). The study carried out through social media such as *Instagram* was divided into two parts. At first, the profile of a Spanish winter sports federation (FADI, @fadiandalucia) was used; a profile with consolidated followers, which allowed the survey to have a greater reach with more than 1,000 participants compared to the more than 800 participants of the @*mmhealthscience* profile, created during the study period as a scientific knowledge transfer academic platform designed by our nutritional research group in the University of Granada, Spain (3): the age of the population, mostly young adult people (up to 55 years old), the majority profile in the use of social media. This population coincides with that used in a similar study by Mariscal-Arcas (3).

Analysing the results inferred in the study, we can deduce information about the food consumption of our population and also the state of mind during the situation experienced in the days of confinement. In general, it can be seen that the consumption of dairy products, fruits, vegetables, legumes, and eggs is quite high throughout the confinement and linear (it remains high at all times, except in the case of fruits, vegetables, and legumes that rises as the progress of study days). It could be because the study was carried out in a Spanish population, almost entirely Andalusian Region and Murcia Region, so that the MD is very present in daily life, and it is not affected by confinement (neither increasing nor decreasing). However, along the same lines as the Mediterranean pattern, it should be noted that the consumption of animal protein sources, such as meat and fish, increases throughout the quarantine period with no differences between gender as described by Tkachenko (25). Therefore, perhaps influenced by having more time to devote to cooking, a consequence of the restriction of the population's freedom of movement seems that more time has been spent making more sophisticated and different recipes, trying new foods, etc. This theory is maintained in other studies, carried out during the pandemic, such as the one carried out by Di Renzo in the Italian population. In that, the increase in the consumption of natural products to the detriment of processed ones, an increase in the preparation of meals, and a greater adherence to the Mediterranean pattern are defended although it was already high (26). It could also be due to a greater concern for health these days where the fact of maintaining an adequate state of health acquires special relevance. This theory of greater self-care for health would be supported by Luzi and Radaelli who speak of being overweight as the main risk factor not only because it increases the risk of infection and complications for obese people, but also a high prevalence of obese individuals within the population, and it may increase the chance of the emergence of a more virulent viral strain, prolong the shedding of the virus throughout the population, and could eventually increase the overall death rate from a flu pandemic (27).

It could also be due to a greater concern for health these days where the fact of maintaining an adequate state of health acquires special relevance. This theory of greater self-care for health would be supported by Luzi and Radaelli (27) who speak of being overweight as the main risk factor not only because it increases the risk of infection and complications for obese people, but also a high prevalence of obese individuals within the population, and it may increase the chance of the emergence of a more virulent viral strain, prolong the shedding of the virus throughout the population, and could eventually increase the overall death rate from a flu pandemic along these lines of increased health concern, one could speak of the reduction in alcohol consumption declared, with higher consumption in the middle of the period. According to the study carried out by Sidor (28), alcohol consumption in some people is increased due to the stress produced by prolonged stays at home, although it only accounts for 14% of the study population (28). However, in this case, no statistically significant correlation is observed according to Pearson's correlation analysis, therefore, confinement would have nothing to do with the consumption or not of alcoholic beverages. It is a stable habit in the population that it could be said that is not affected by the stress that a confinement situation can entail or the uncertainty of not knowing what will happen (work situation, evolution in the number of cases of infected by SARS-CoV-2) or the idea of drinking to escape. In addition, the consumption of ‘junk food' such as snacks, industrial pastries, sweets, and soft drinks is also reduced over time and also ‘snacking' between meals. In both cases, there is a significant negative correlation (97%), that is, as the days of confinement go by, the study population decreases the consumption of this type of products and also snacking, a theory confirmed by Di Renzo (26).

Water consumption does present an upward trend line throughout the confinement, although from the beginning, the percentage of YES responses remains high, with a daily average that does not fall below 90% with a positive correlation and a significance level of 97%, so the passage of days would be related to the increase in water consumption. We could also relate it to an increase in the subjects' concern for the state of health (19, 29). A recent review highlights that balanced nutrition can help maintain immunity, which is essential for the prevention and treatment of viral infections (30). We can conclude that the period of confinement due to the COVID-19 pandemic in Spain has induced changes in diet with a trend toward a higher consumption of fruit, vegetables, pulses, and fish and a lower consumption of bakery products, sweets, salty snacks, sugary drinks, and drinks with a high alcoholic content as other authors have found (19).

The reduction in physical activity to which the population has been subjected could have caused a greater sensation of heaviness on the part of the subjects; however, according to the data obtained from the results of the study, it does not seem to have influenced since it remains fairly stable throughout the days and does not present a significant correlation. The mood question of this study improves as the days go by, going from a daily average of 45% affirmative responses to 55% toward the end of confinement. It could be related to the end of the confinement situation (although the state of alarm is maintained, prohibitions are being lifted and they begin to let us go outside) although it could also be thought that it may be due to an adaptation to the new situation. In this last line, it is possible to establish a relationship with the state of imprisonment of criminals serving a sentence. Echeverri (31) analyses the psychological effects and the evolution of prisoners and establishes that, contrary to what was thought, as life in freedom is closer, the inmate manifests a greater conformity with the established social norms. The daily reality is imposed since the person who has been confined in prison for many years, as he sees the possibility of reintegrating himself into life in freedom, progressively adapts his behaviour to the social norms that he himself transgressed (31). However, the data referring to the correlation between both variables (passage of days and mood question) were not positive. According to Pearson's correlation analysis, there is no relationship between variables, so none of the theories outlined above would be valid. There is a good acceptance by the population regarding the daily post that is published since it began with 80% of YES responses with a slight rise over the days. Communicating nutrition information is a good strategy to reduce boredom, stress, and anguish caused by having to stay at home (3, 32); however, if the correlation between variables in Table 3 is analysed, there is no significant correlation, so it can be said that publishing publications on a daily basis does not impact so much over time. Therefore, perhaps it would be more interesting to select good information with the intention of having a great impact but more sporadically.

The nutritional health information through the social media claim to improve the health status of the participants. Other studies have found that the influence of the confinement situation due to COVID-19 may have on the use of the Internet as a source of information on health-related issues in the world population. This situation of confinement could have affected the perception, the feeding, and the use of social media (3). The diffusion through social media has allowed to have a greater reach of the population, including different parts of Spain that are distant from each other and, for the most part, the young adult population. We observed that mood throughout confinement generally improves, although it is not related to the passage of time in confinement. There are certain eating habits from the MD that are well established in the daily diet of our population, such as the consumption of fruits, vegetables, legumes, dairy products, and eggs. It seems that enjoying good health is a growing concern in pandemic situations, which is why inappropriate behaviours such as “snacking” between meals or the consumption of processed foods such as snacks, industrial pastries, soft drinks, and sweets are avoided, increasing the amount of healthy food such as meat and fish. Future studies should examine that the effect these habits may have in quarantine situations. Finally, we conclude that the daily publication of posts is not as impressive sustained over time compared to if articles of higher quality of content are released more sporadically. This study, as indicated by Mariscal-Arcas (3), opens up future avenues of research promoting MD and implements new cohort nutritional databases, especially about young adult people, who are adept at navigating digital spaces and therefore using social media (24, 33–38).

## Data Availability Statement

The original contributions presented in the study are included in the article/supplementary material, further inquiries can be directed to the corresponding author.

## Ethics Statement

The studies involving human participants were reviewed and approved by Research Ethics Committee of the Andalusian Public Health Service, Spain. Written informed consent to participate in this study was provided by the participants' legal guardian/next of kin.

## Author Contributions

The study was designed by MM-A. Data were collected and analysed by MM-A, SD-M, BS, AB-D, JL, MM-B, NG-B, JC-P, LC, AL-M, and MJ-C. Data interpretation and manuscript preparation were undertaken by MM-A, JC-P, LC, AL-M, and MJ-C. All authors contributed to the article and approved the submitted version.

## Funding

This study was supported by the Andalusian Regional Government (Nutrition, Diet and Risks Assessment: AGR255), FEDER-ISCIII PI14/01040 and Consejería de Transformación Económica, Industria, Conocimiento y Universidades, Junta de Andalucía P18-RT-4247.

## Conflict of Interest

The authors declare that the research was conducted in the absence of any commercial or financial relationships that could be construed as a potential conflict of interest.

## Publisher's Note

All claims expressed in this article are solely those of the authors and do not necessarily represent those of their affiliated organizations, or those of the publisher, the editors and the reviewers. Any product that may be evaluated in this article, or claim that may be made by its manufacturer, is not guaranteed or endorsed by the publisher.
